# Cardiovascular complications in a diabetes prediction model using machine learning: a systematic review

**DOI:** 10.1186/s12933-023-01741-7

**Published:** 2023-01-19

**Authors:** Ooi Ting Kee, Harmiza Harun, Norlaila Mustafa, Nor Azian Abdul Murad, Siok Fong Chin, Rosmina Jaafar, Noraidatulakma Abdullah

**Affiliations:** 1 UKM Medical Molecular Biology Institute (UMBI), Universiti Kebangsaan Malaysia (UKM), 56000 Kuala Lumpur, Malaysia; 2 Department of Medicine, Faculty of Medicine, Universiti Kebangsaan Malaysia (UKM), 56000 Kuala Lumpur, Malaysia; 3 Faculty of Engineering and Built Environment, Universiti Kebangsaan Malaysia, 43600 Bangi, Malaysia; 4 Faculty of Health Sciences, Universiti Kebangsaan Malaysia (UKM), 50300 Kuala Lumpur, Malaysia

**Keywords:** Type 2 diabetes mellitus, Cardiovascular disease, Machine learning, Prediction model

## Abstract

Prediction model has been the focus of studies since the last century in the diagnosis and prognosis of various diseases. With the advancement in computational technology, machine learning (ML) has become the widely used tool to develop a prediction model. This review is to investigate the current development of prediction model for the risk of cardiovascular disease (CVD) among type 2 diabetes (T2DM) patients using machine learning. A systematic search on Scopus and Web of Science (WoS) was conducted to look for relevant articles based on the research question. The risk of bias (ROB) for all articles were assessed based on the Prediction model Risk of Bias Assessment Tool (PROBAST) statement. Neural network with 76.6% precision, 88.06% sensitivity, and area under the curve (AUC) of 0.91 was found to be the most reliable algorithm in developing prediction model for cardiovascular disease among type 2 diabetes patients. The overall concern of applicability of all included studies is low. While two out of 10 studies were shown to have high ROB, another studies ROB are unknown due to the lack of information. The adherence to reporting standards was conducted based on the Transparent Reporting of a multivariable prediction model for Individual Prognosis or Diagnosis (TRIPOD) standard where the overall score is 53.75%. It is highly recommended that future model development should adhere to the PROBAST and TRIPOD assessment to reduce the risk of bias and ensure its applicability in clinical settings. Potential lipid peroxidation marker is also recommended in future cardiovascular disease prediction model to improve overall model applicability.

**Supplementary Information:**

The online version contains supplementary material available at 10.1186/s12933-023-01741-7.

## Introduction

Machine learning is a branch of computer science that uses existing data to predict future responds when new data is provided [1]. By utilizing artificial intelligence, pattern recognizing, and computational statistics, the training of prediction model can improve its overall performance and make decisions based on new set settings or situations.

Early prediction of cardiovascular disease (CVD) among diabetes patients were created based on logistic regression, but machine learning has been used as a predictive model for its flexibility and variability [2]. Regression models are made based on a hypothesis and a fixed model structure but machine learning search for the optimal fit based on different algorithms [3]. Various machine learning algorithms are used in creating predictive model such as neural networks (NN), support vector machine (SVM), decision tree (DT), and *k*-nearest neighbours (*k-*NN) [4]. The building of predictive model using machine learning approach will require extra steps that include the model training and validation. Through repeated training and testing of models, different algorithms can only be compared among each other to find out the best performing model or algorithm.

The performance of machine learning model also affected by the predictors or risk factors used in the model [5]. Several risk factors that involve in the development of atherosclerosis which lead to CVD in individuals with T2DM were include hypertension, insulin resistance, hyperglycaemia, obesity, and dyslipidaemia [6]. In addition, recent studies have shown that T2DM patients have higher risk in developing CVD due to lipid peroxidation where free radicals or reactive oxygen species (ROS) attacked polyunsaturated fats (PUFAs) [7, 8]. Polyunsaturated acyl group of phospholipids lose its hydrogen to form a highly reactive radical, followed by the reaction with oxygen to form a peroxyl radical [9]. The peroxyl group is then obtain hydrogen from other phospholipids to form a lipid hyperperoxide [10]. The peroxide will react with other organic substrate such as another phospholipid [11]. As the result, production of electrophilic molecules such as malondialdehyde (MDA) increases causing oxidative stress [12]. The cytotoxicity of these molecules can cause complications such as aging and atherosclerosis by binding to DNA, proteins, or other nucleophilic molecules. These damages induce cell death and eventually progress into cardiovascular complications [11]. Since the increase of level of lipid peroxide molecules and oxidative stress known for causing CVD, the level of these biomolecules can be potentially used as predictors in the development of prediction model for cardiovascular disease among diabetes patients.

Previous studies have provided the basis to build a disease prediction model by machine learning [13]. Machine learning (ML) approach offers the opportunity to identify patients at greater risk of T2DM complications [14] while prediction models built using ML techniques improve cardiovascular disease prediction and reducing the number of screenings required when compared with the ACC/AHA Pooled Cohort Equations (PCE) calculator alone [15].

In the Action to Control Cardiovascular Risk in Diabetes Study (ACCORD) and the Veterans Affairs Diabetes Trial (VADT) trials, a ML analysis provided evidence supporting the diabetes treatment guideline recommendation of intensive glucose lowering in diabetes patients with low cardiovascular risk and additionally suggested benefits of intensive glycaemic control in some individuals at higher cardiovascular risk [16]. Moreover, an unsupervised ML clustering method could address T2DM patients with heterogeneous clinical indicators and identify groups with different types of coronary plaque and degrees of coronary stenosis, allowing patient stratification [17]. In addition, a ML approach demonstrated high performance in identifying metabolic-associated fatty liver disease (MAFLD) patients with prevalent cardiovascular disease based on the easy-to-obtain patient parameters [18]. Finally, incorporating genome-wide polygenic risk score (gPRS) and serum metabolite data enhances diabetes risk prediction [19]. The application of the cardiovascular diabetes prediction model can assist in clinical settings such as decision-making, clinical management in diabetes care, and patient communication to reduce the risk of cardiovascular complications among diabetes patients [20]. In this context, this systematic review is to identify the available machine learning-based prediction models for diabetic cardiovascular disease.

## Methods

Systematic searches and the development of this review was guided by Preferred Reporting Items for Systematic Review and Meta-analyses Protocols (PRISMA-P) [21]. A protocol has been registered at The International Prospective Register of Systematic Reviews (PROSPERO) under the reference ID CRD42022337764. Criteria of the studies are outlined based on PICOTS framework [22].

### Participants (P)

Studies that involve patients that are diagnosed with T2DM. These patients include individuals with or without cardiovascular complications. Studies that involve other types of diabetes were not included in this review. There were no eligibility restrictions on age, population, gender, ethnicity, geographic location of participants. Studies that include other diabetes complications are included.

### Interventions (I)

Only predictive modelling studies that clearly describe the use of machine learning (ML) in prognosis and diagnosis models are included. Therefore, studies without the clear demonstration of ML-based prediction model will be excluded. Studies that include supervised, unsupervised or combination of both are accepted as the interventions to build a predictive model. Since different studies’ aim may or may not require external validation, thus, the three study types that are, prediction models development studies without external validation, prediction model development studies with external validation, and external model validation studies with or without model updating were included [23].

### Outcomes (O)

The effects and properties of prediction models were observed and measured in this review based on the reported metrics, including c-statistics or classification measurements such as, the accuracy, sensitivity, and specificity. Secondary outcomes that were observed were study design, population, predictors, and model types.

### Time (T)

The search was limited to publications from 1st January 2017 to 14th April 2022 to ensure data were up to date within five years from this study.

### Settings (S)

Only studies published in English were included.

### Search strategies

A uniform systematic search was performed in two databases including SCOPUS and Web of Science. Relevant articles from the references were searched manually. The search terms are based on the PICOTS list in Table 1.

**Table 1:** Selection criteria of predictive modelling studies in PICOTS format

	Participants (P)	Intervention (I)	Comparison (C)	Outcomes (O)	Timeframe (T)	Settings (S)	Other limitations
Inclusion criteria	Patients with T2DM	ML-based predictive modelling including supervised and unsupervised machine learning or combination of both	N/A	Study designs, population, predictors, and models used, quality validation of models	From 1st January 2017 to date	N/A	Language = English
Exclusion criteria	Patients with other types of diabetes or pre-diabetes	Prediction models without specific use of ML					

### Data extraction and risk of bias assessment

The extraction had been performed according to the Transparent Reporting of a multivariable prediction model for Individual Prognosis or Diagnosis (TRIPOD) statement [24, 25]. TRIPOD statement had also been used for reporting adherence. ROB for each studies included was carried out based on Prediction Model Risk of Bias Assessment Tool (PROBAST) [26] (Additional files 1, 2).

## Results

**Fig. 1:**
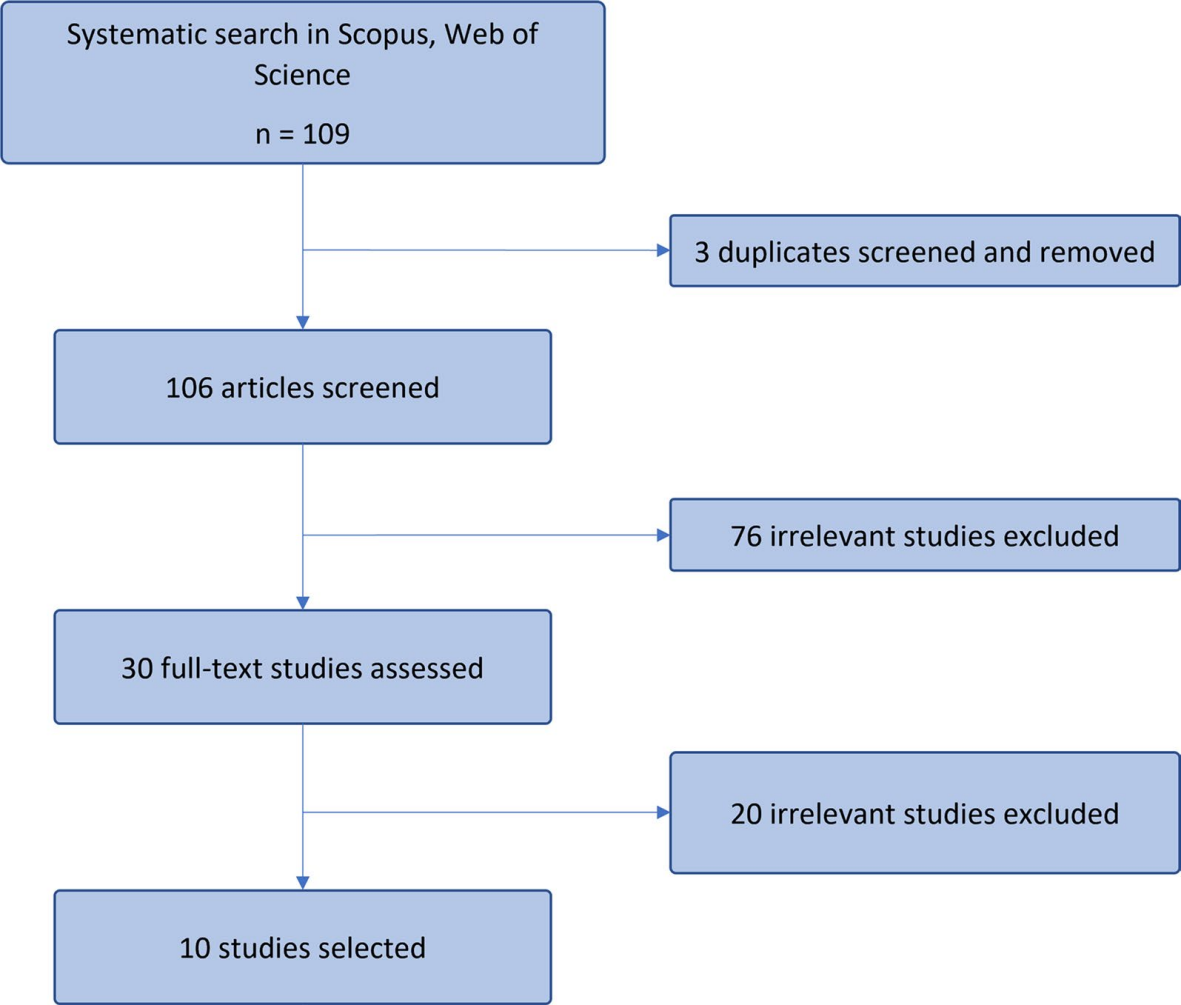
PRISMA flow diagram for the inclusion of cardiovascular diabetes prediction models from 1st January 2017 till 14th April 2022

Of 109 articles reviewed, only 10 were selected in this review article (Fig. 1). The general characteristics of each included study are described in Table 2. All studies included in this review were cohort studies all over the world except for only one is from cross-sectional study that conducted in China (Table 2). Majority of the studies were based on the European/Caucasian population (Germany, Greece, Sweden, Denmark, Australia, and United States), but only two studies were based on the Chinese population.

**Table 2:** General characteristics of the included studies in the systematic review of cardiovascular diabetes prediction models

References	Study designs	Population	Predictors	Model types	Outcome
[31]	Cohort	Greece	Body mass index (BMI), Hba1c, fasting blood glucose (FBG), lipid profile (LP), age, smoking habit, hypertension (HPT), pulse pressure, lipid-lowering therapy, parental history of diabetes	XGBoost	Development of prediction model for fatal or non-fatal incidence in T2DM individuals
[32]	Cohort	Denmark	Disease codes, prescription of insulin and analogues, and prescription of blood glucose lowering drugs	Logistic ridge regression, random forest, decision tree gradient boosting	Prediction of individuals at elevated risk of developing T2DM comorbidities
[29]	Cohort	Greece	Age, diabetes duration, Hba1c, blood pressure (BP), FBG, LP, smoking habit, sex, HPT, lipid-lowering therapy	HWNN, SOM, BLR, FFN, CART, RF, NB	Development of prediction model for fatal or non-fatal incidence in T2DM individuals
[30]	Cohort	United States	BMI, BP, age, sex, hypertensions, heart and diabetic complications, other nosology, insulin, sugar-lowering drugs, other drugs	XGBoost, DT, RF, LR, Dummy, *k*NN, multinominal, complement and Bernoulli’s NB	Prediction of individuals at elevated risk of developing T2DM comorbidities
[13]	Cohort	United States, Greece	Age, diabetes duration, BMI, BP, Hba1c, FBG, LP, HPT, ACE inhibitor, sex, diabetic parents, retinopathy, calcium antagonists, diuretics, B-blockers, smoking habit, proteinuria, hypolipid diet, aspirin, diet, sulphonyl urea, diguanide, insulin	ANN, binary logistic model, logistic model tree, Bayes net, DT, naïve Bayes	Assess the ability and performance of six machine learning models in prediction T2DM and CVD complications
[33]	Cohort	China	Sex, age, race, total cholesterol, high density lipoprotein (HDL), systolic BP, anti-HPT treatment, diabetes, and smoking habit	Knowledge learning symbiosis (KLS)	Development of prediction model for CVD risk in T2DM individuals
[34]	Cohort	Sweden	Sex, systolic BP, BMI, smoking habit, diagnosis of atrial fibrillation, myocardial and stroke history, HbA1c, HDL, total cholesterol, duration of type 2 diabetes, microalbuminuria, macroalbuminuria	Cox gradient boosting machine learning (GBM)	Assess eighty cardiovascular and inflammatory proteins for biomarker discovery and the prediction of major cardiovascular events in T2DM
[28]	Cross-sectional	China	Sex, age, marital status, educational level, monthly income, diabetes duration, insulin treatment, HbA1c, FBG, LP, BP, BMI, anxiety, depression, smoking habit, and drinking habit	Deep neural network	Development of a CVD risk prediction model based on the bio-psycho-social contributors in T2DM patients
[35]	Cohort	Australia	Age, sex, admission episode, discharge dates and disease codes	LR, SVM, DT, RF, NB, *k*NN	Development of prediction model for CVD risk in T2DM individuals
[36]	Cohort	United States	BMI, age, and fasting plasma glucose	SVM, *k*NN	Development of prediction model for CVD risk in T2DM individuals

The most common predictor used in the predictive model was HbA1c, which six out of ten studies included in their model, followed by body mass index (BMI) where 50% used in their model and medical history or disease, which only included in three articles (Table 2). Other predictors were sex, age, heart rate, blood pressure, lipid profile, fasting blood glucose, waist circumference, parental history of diabetes, patients’ smoking or drinking habits and the treatment of the patients received such as insulin treatment and lipid-lowering treatment. Among the 18 predictors involved in this study, the top five predictors are BMI, anxiety, depression, total cholesterol, and systolic blood pressure.

There were several models or algorithms that have been reported to predict cardiovascular diabetes so far such as support vector machine (SVM), decision tree (DT), random forest (RF), Naïve Bayes (NB), linear regression (LR), Self-Organizing Maps (SOM), and knowledge learning symbiosis (KLS) [27]. Gradient boosting models have been reported in four studies that included extreme gradient boosting, cox gradient boosting, and decision tree gradient boosting. Other reported models were neural networks and k-nearest neighbour from another three studies (Table 2).

### Model performance

From the review, not all studies reported their model performance using the same metrics of evaluation. Based on Table 3, neural network model [28] has the best performance which achieve 87.5% accuracy, 88.06% sensitivity, 87.23% specificity and AUC of 0.91. The precision of the model was not reported but based on the confusion matrix provided, it is 76.6%. In addition, previous studies [13, 28, 29] have shown that the overall performance of neural network is better than gradient boosting.

**Table 3:** Performance of the proposed models reported using various metrics of evaluation including accuracy, sensitivity, specificity, precision, C-value, and area under the curve

References	Best performing model	Accuracy (%)	Sensitivity (%)	Specificity (%)	Precision (%)	Area under the curve
[31]	XGBoost	NA	71.00 ± 23.85	NA	NA	71.13 ± 11.69
[32, 34]	Gradient Boosting Machine	NA	79.1	55.8	NA	0.69–0.825
[29]	Hybrid Wavelet Neural Network (HWNN)	83.04 ± 8.22	29.50 ± 23.15	87.30 ± 9.73	NA	67.64 ± 15.09
[30]	XGBoost	84.5	85	NA	84.5	NA
[13]	Ensembles of ANN	80.20	NA	NA	NA	0.849
[33]	Knowledge Learning Symbiosis (KLS)	NA	NA	NA	NA	NA
[28]	Neural network	87.50	88.06	87.23	76.6	0.91
[35]	Logistic regression, support vector machine	83.33	83.33	83.33	83.33	0.81
[36]	Support vector machine	96.93	92.87	NA	94.44	NA

Gradient boosting algorithm was indicated as the second-best performing algorithms after neural network based on the performance metrics provided. A cohort study conducted in US [30] has shown gradient boosting with the highest performance with 84.5% accuracy, 85% sensitivity, and precision of 84.5% compared to other models. This is supported with previous study conducted in Greece [31] that extreme gradient boosting (XGBoost) has the potential to handle the imbalanced medical dataset. In that study, the best reported model was based on XGBoost with sensitivity of 71.00% (CI 74.15, 94.85) and AUC of 0.71 (CI 0.59, 0.83). The third performing model are the only comparable models with complete performance data are LR and SVM models [35]. The two models in this study have the same performance with 83.33% accuracy, 83.33% sensitivity, 83.33 specificity, 83.33% precision, and AUC of 0.81. SVM model developed in Swedish cohort [36] also reported to perform better compared to *k*-nearest neighbour with 96.93% accuracy, 92.87% sensitivity, and 94.44% precision.

### Risk of bias assessment

**Fig. 2:**
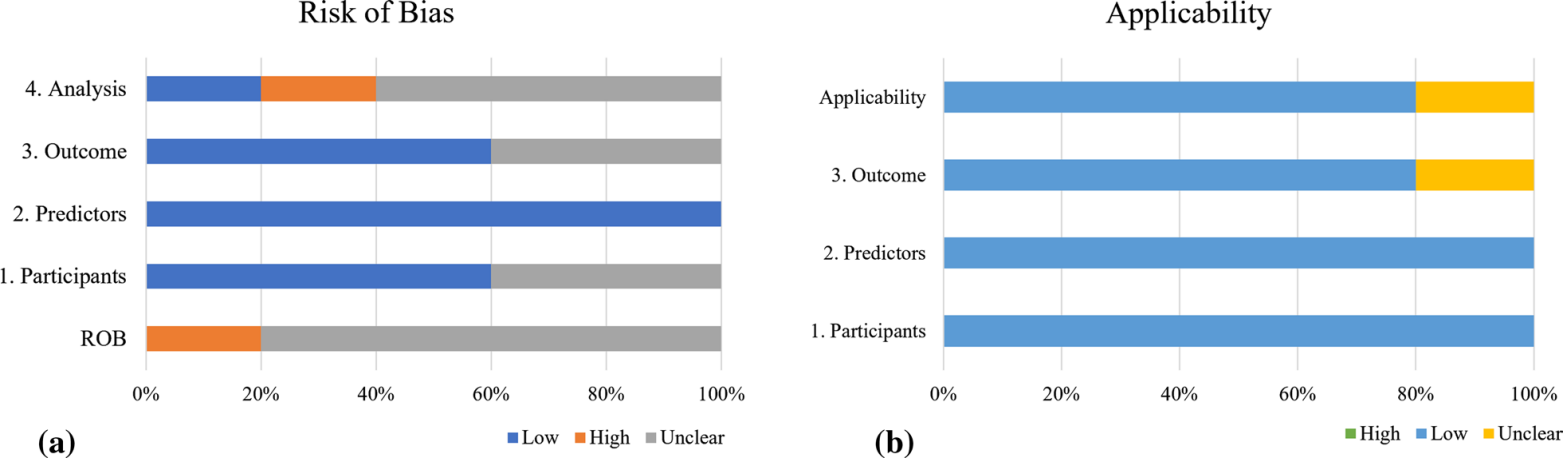
Prediction Model Risk of Bias Assessment Tool (PROBAST) for the studies included in this review. **a** The risk of bias of the 10 included studies. **b** The applicability of the 10 included studies

From Fig. 2, two out of 10 included studies have high risk-of bias, another eight have unclear risk-of-bias. The elevated risk is from the participant domain, which the inclusion and exclusion criteria are often not reported. All included studies have low risk in the predictor’s domain. As in the outcome domain, all studies have minimal risk, while another four studies have an unclear risk due to the lack of information about the time interval between predictor assessment and outcome determination. Whereas another six studies have unclear risk in their analysis due to the lack of multivariable analysis and four studies did not discuss about sampling controls. Although there is no study with low overall risk of bias, the concern of applicability for all models developed are low because the included participants and settings, definition, timing, or assessment of predictors, and the outcome definition, timing, or determination in all studies match the review question.

### Adherence to reporting standards

**Fig. 3:**
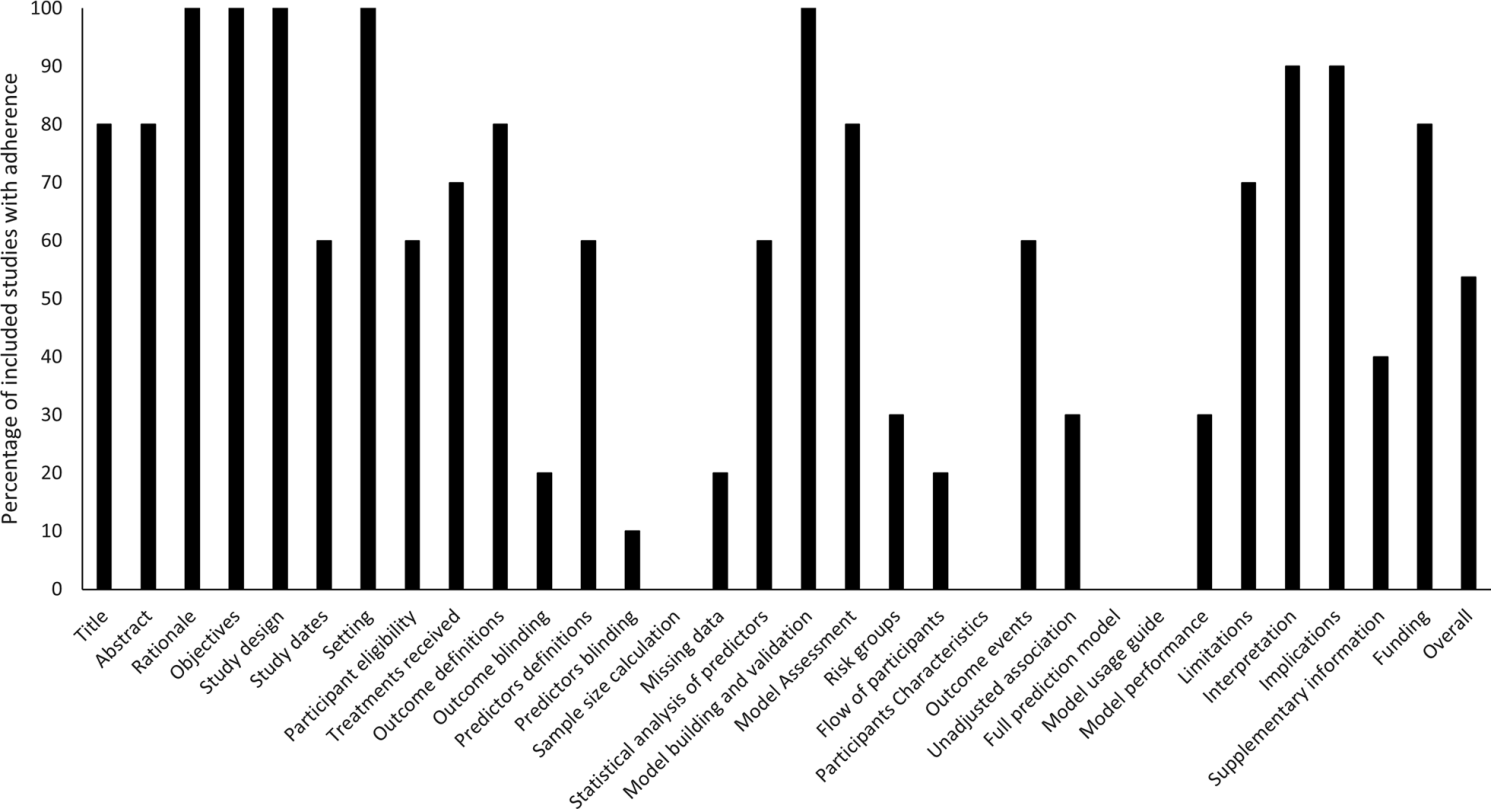
Adherence of included studies to Transparent Reporting of a Multivariable Prediction Model for Individual Prognosis or Diagnosis (TRIPOD) assessment

The overall percentage of adherence to reporting standards based on the TRIPOD assessment is 53.75% with 13 out of 31 items with less than 50% adherence (Fig. 3). However, there are four out of the 13 items have 0% of adherence, which are sample size calculation, participant characteristics, full prediction model, and model usage guide. Other items with less than 50% reporting adherence are outcome blinding, predictors blinding, missing data, risk groups, flow of participants, unadjusted association, and model performance. The rationale, objectives, study design, setting, and model building and validation have a 100% reporting adherence. The lack of reporting adherence about full prediction model and model usage rendered the model unapplicable in real life clinical settings (Table 4).

**Table 4:** PROBAST results

Study	ROB				Applicability			Overall	
	Participants	Predictors	Outcome	Analysis	Participants	Predictors	Outcome	ROB	Applicability
Athanasiou et al. 2020	−	+	+	+	+	+	+	−	+
Dworzynski et al. 2020	+	+	+	−	+	+	+	−	+
Zarkogianni et al. 2018	−	+	+	?	+	+	+	−	+
Derevitskii and Kovalchuk 2020	+	+	+	?	+	+	+	?	+
Dalakleidi et al. 2017	?	+	+	?	+	+	+	?	+
Mei and Xia 2019	+	+	+	?	+	+	+	?	+
Nowak et al. 2018	+	+	?	+	+	+	+	?	+
Chu et al. 2021	+	+	?	−	+	+	+	−	+
Hossain et al. 2021	+	+	?	?	+	+	+	?	+
Miao et al. 2020	?	+	?	?	+	+	+	?	+

“+” indicates low ROB/low concern regarding applicability; “−” indicates high ROB/high concern regarding applicability; and “?” indicates unclear ROB/unclear concern regarding applicability

*ROB* risk of bias

## Discussion

This review identified ten machine learning models that were developed for predicting cardiovascular disease among diabetic patients conducted mostly among European population. Even though the prevalence of cardiovascular diabetic was high in Asian countries, only two included studies were conducted among the Chinese population but none from the Malay or Indian population. In 2019, 44.2% of Malaysian patients presented with acute coronary syndrome had diabetes which is the second common cardiovascular risk factor (CVRF) after hypertension (61.9%) [37]. Thus, this highlighted the importance of conducting predictive model studies of diabetic cardiovascular disease for Malaysian since its population also pose a higher risk at younger age than the European population [38]. Furthermore, a sharp increase of T2DM treatment cost from USD 232 billion in 2007 to USD 966 billion in 2021 with the high prevalence of the disease worldwide is causing concerns on its burden in the lower-income nations [39]. It is known that most T2D patients do not require insulin for the rest of their life, but the complications developed from T2D eventually increase the economic burden on the patients and the healthcare system worldwide [1, 40]. Thus, it is essential to develop cardiovascular diabetes prediction model to effectively reduce the morbidity and further complication as well as the economic burden especially in Asian countries.

The artificial neural network model (ANN) reported by Dalakleidia and Zarkogianni [13] showed that ANN performed better than other algorithms such as NB, decision tree, and logistic model when working with the imbalanced nature of medical datasets. Imbalanced dataset is when the distribution of classes is unequal that leads to the situation where one class out represent the other. This suggest that receiver operating curves, precision-recall curves, and cost curves are necessary when imbalanced datasets are involved [41]. This nature of medical datasets lead to prediction bias towards larger disjuncts and misclassification of the smaller disjuncts [42]. To avoid class imbalance, oversampling of the minority class such as the use of Synthetic Minority Oversampling Technique (SMOTE) can help improving the overall accuracy of a model [14]. In addition, three out of ten studies in this review have supported that neural network can be used to construct predictive models for diabetic cardiovascular disease. However, the three studies that involve neural network did not include the use of gradient boosting algorithm, thus, these models were not compared based on their accuracy, sensitivity, specificity, and precision.

Before machine learning was introduced, prediction models were developed using classical statistics such as logistic regression. The Framingham Heart Studies (FHS) is one of the most famous examples of a prediction model for cardiovascular disease that applies logistics regression [43] and the focus on diabetes mellitus as a risk factor of CVD emerged after years of follow up studies [44, 45]. Other than logistic regression, a classic statistical model such as the cox regression model was also applied in the development of CVD prediction model for diabetic patients. For example, a study that incorporated the patient population and electronic medical record (EMR) data in US [46] developed a cox regression model with a c-statistic of 0.782 and the model reported in Ley et al. [47] achieved a c-statistic of 0.73 (0.72–0.74). While classical statistic has been applied in various medical disciplines from CVD to cancer studies [48–50], machine learning model is advantageous when working with pattern recognition other than just projection based on existing data [51].

Predictors in a predictive model are important as it affects the performance when dealing with new datasets. However, not all studies mention about the impact of the predictors involved in their models. Out of ten articles reviewed, only four studies summarized the most important factors for their model and BMI were reported as the top five key factors. BMI has been used as an obesity indicator, which is directly linked to cardiovascular disease and diabetes [52]. With the increasing availability of fast food and processed food, the general eating habit and diet of most people are known to become less healthy due to increasing carbohydrates and fat intake. This phenomenon worsens in recent years as part of the urbanization [53]. Body mass index reflects the diet of an individual which is also known to be a strong factor in causing cardiovascular complications among diabetic patients [54]. Although its contribution to the prediction models was not reported in all included studies, six out of ten articles used BMI as one of their predictors. Although family history is known to be a major risk factor in the development of the CVD, but none of the study included this predictor. This might be due that family history of CVD has been excluded in these studies [55, 56]. In the past few decades, the number of studies on lipid peroxidation is increasing due to its association with cardiovascular disease through lipid alteration [8, 57]. The earliest mention of lipid peroxidation with extensive discussion is in 1958 by Lundberg [58]. Since then, more studies about the autoxidation of lipid were published. Even though the relationship between increased lipid peroxidation level in diabetic patients and risk of CVD is well known [59], no studies included in this systematic review included any lipid peroxidation marker as a predictor in model development.

Although this review is quite comprehensive that followed the guideline of PRISM-P, the selection framework by PICOTS and addressed all the risk assessment bias using PROBAST and TRIPOD, but the search of this study was only performed on Scopus and WoS only. Meta-analysis also could not be done due to the limited number of articles and recent studies. To address the imbalanced nature of clinical data, which is very common in life sciences, precision-recall curve (PRC) is the recommended metric to display the true performance of a prediction model [60]. The sample size varies significantly among the prediction models discussed in this review, ranging from 560 in the study by Nowak and Carlsson [34] to more than 200,000 subjects in the study by Dworzynski, Aasbrenn [32]. The best model has the sample size of 834 subjects where the study with second best models involved 124,000 subjects.

For clinical practice, prediction models are required to be user friendly, and the presentation of the results also play a vital role in the communication between clinician and patients. Furthermore, to ensure the reliability and overall precision of a prediction model, external validation must be conducted using new datasets as the test data [61]. In the future, existing models can also be improved using newly collected data.

## Conclusion

In this review, we discovered ten studies of cardiovascular disease prediction models among T2DM patients which used various machine learning approaches. The best model among the studies is the neural network model proposed by Chu and Chen [28] with AUC of 0.91. However, the precision of the model is only 76.6% and external validation is recommended to verify its performance when dealing with different datasets. External validation is a crucial step to ensure the applicability of a model in clinical settings [14]. This review shows that neural network has the best performance followed by gradient boosting machine to predict cardiovascular disease among diabetes patients. Future studies are recommended to include the comparison between neural network and gradient boosting machine using same datasets.

## Supplementary Information

**Additional file 1.** PROBAST assessment form.

**Additional file 2.** TRIPOD assessment form.

## Data Availability

Not applicable.

## Abbreviations

BP: Blood pressure
BMI: Body mass index
CVD: Cardiovascular disease
CVRF: Cardiovascular risk factor
FHS: Framingham Heart Studies
LR: Linear regression
SVM: Support vector machine
RF: Random forest
KLS: Knowledge learning symbiosis
AUC: Area under the curve
ML: Machine learning
PRC: Precision-recall curve
T2DM: Type 2 diabetes
